# Visualization of the lymphocytic choriomeningitis mammarenavirus (LCMV) genome reveals the early endosome as a possible site for genome replication and viral particle &nbsp;pre-assembly

**DOI:** 10.1099/jgv.0.000930

**Published:** 2017-09-27

**Authors:** Benjamin R. King, Samuel Kellner, Philip L. Eisenhauer, Emily A. Bruce, Christopher M. Ziegler, Daniel Zenklusen, Jason William Botten

**Affiliations:** ^1^​ Department of Medicine, Division of Immunobiology, University of Vermont, Burlington, VT 05405, USA; ^2^​ Cellular, Molecular, and Biomedical Sciences Graduate Program, University of Vermont, Burlington, VT 05405, USA; ^3^​ Departement de Biochimie et Médecine Moléculaire, Université de Montréal, Montréal, QC H3T 1J4, Canada; ^4^​ Department of Microbiology and Molecular Genetics, University of Vermont, Burlington, VT 05405, USA

**Keywords:** arenavirus, LCMV, RNA FISH, viral replication complex, Rab5c, viral RNA genome

## Abstract

We report a fluorescence *in situ* hybridization (FISH) assay that allows the visualization of lymphocytic choriomeningitis mammarenavirus (LCMV) genomic RNAs in individual cells. We show that viral S segment genomic and antigenomic RNA, along with viral nucleoprotein, colocalize in subcellular structures we presume to be viral replication factories. These viral RNA structures are highly dynamic during acute infection, with the many small foci seen early coalescing into larger perinuclear foci later in infection. These late-forming perinuclear viral RNA aggregates are located near the cellular microtubule organizing centre and colocalize with the early endosomal marker Rab5c and the viral glycoprotein in a proportion of infected cells. We propose that the virus is using the surface of a cellular membrane-bound organelle as a site for the pre-assembly of viral components, including genomic RNA and viral glycoprotein, prior to their transport to the plasma membrane, where new particles will bud.

## Abbreviations

Cy3, cyanine 3; Cy5, cyanine 5; FISH, fluorescence in situ hybridization; GPC, glycoprotein precursor; p.i., post-infection; LCMV, lymphocytic choriomeningitis virus; MTOC, microtubule organizing centre; NP, nucleoprotein.

## Full-Text

**Fig. 1.**
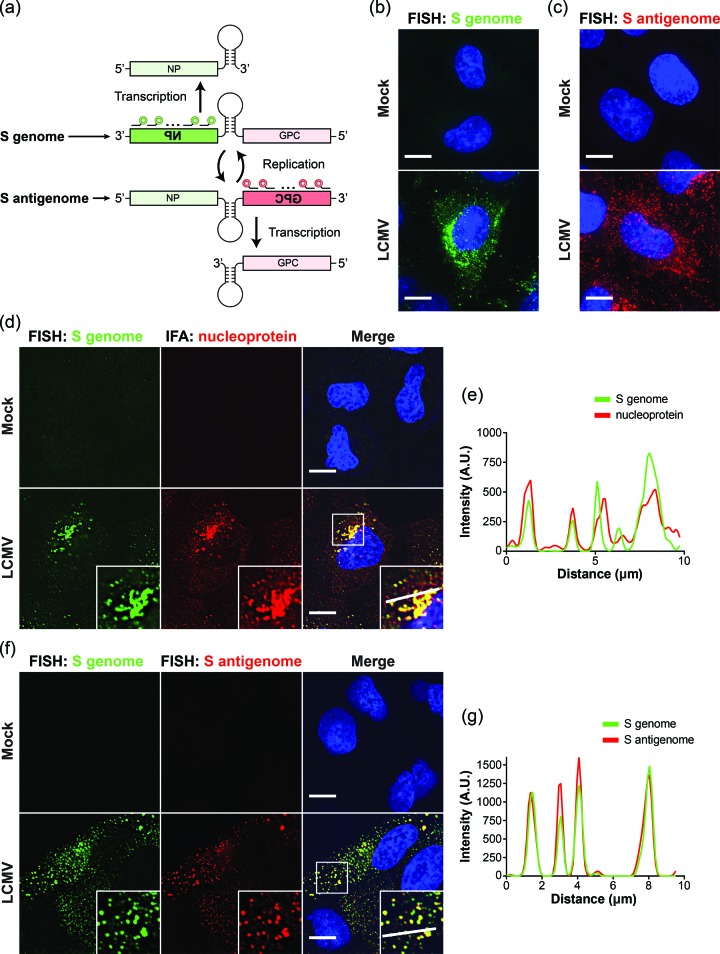
Visualization of S genome and antigenomic RNAs by multiple, singly-labelled FISH probes. (a) Diagram showing the transcription and replication scheme of the LCMV S genomic RNA. Briefly, the S genome serves as the template for the viral polymerase to generate full-length, antigenome replicative intermediates. The S genome and S antigenome serve as templates for the transcription of the NP and GPC mRNAs, respectively. FISH probe sets (each containing 48 individual 20mer probes bearing a single fluorophore at their 3′ terminus) were used to specifically visualize either the S genomic or S antigenomic RNA. (b) Maximum intensity projection of either mock- or LCMV-infected cells (48 h p.i.) stained with S genome FISH probes labelled with Cy3. (c) Maximum intensity projection of either mock- or LCMV-infected cells (48 h p.i.) stained with S antigenome FISH probes labelled with Cy3. (d) Single Z stack of either mock- or LCMV-infected cells (48 h p.i.) stained for S genome (Cy5) and LCMV nucleoprotein [1–1.3 (from M. Buchmeier, University of California Irvine) (primary antibody) as previously described [26]; goat, anti-mouse AlexaFluor 488 (secondary antibody)]. (e) Fluorescence line scan of S genome and NP signals along the line indicated in the inset of the merged image in (d). (f) Single Z stack of either mock- or LCMV-infected cells (48 h p.i.) stained with S genome (Cy5) and S antigenome (Cy3) FISH probes. (g) Fluorescence line scan of S genome and S antigenome signals along the line indicated in the inset of the merged image in (f). Scale bar=10 µm.

The major events of transcription and replication of the arenavirus genomic RNA are well understood at a population level (Fig. 1a) [1, 2]. However, the technical limitations of Northern blot and quantitative RT-PCR have hindered our ability to examine these processes in individual cells and to visualize these events with subcellular resolution. Recent improvements in fluorescence *in situ* hybridization (FISH) technologies now permit the fluorescent labelling and microscopic visualization of RNA species at the single-cell and single-molecule levels [3]. This labelling strategy, relying on pools of fluorescently labelled 20mer oligonucleotide probes, allows visualization of target RNAs with a high signal-to-noise ratio and exquisite specificity [3]. The replication dynamics of influenza A and Rift Valley fever viruses (an orthomyxovirus and bunyavirus, respectively) have been examined using this RNA FISH labelling strategy, and have revealed subcellular sites of genomic RNA replication and assembly, and/or selectivity of genome recruitment into assembling particles [4–6].

Arenaviruses, like orthomyxoviruses and bunyaviruses, have a single-stranded, segmented, negative-sense RNA genome [7]. Previous work has suggested that the genomic RNA of Tacaribe virus (a New World arenavirus) associates with intracellular membranes [8]. However, fluorescence microscopy visualizing the subcellular distribution of viral RNAs (nonspecifically-labelled with a chemically modified nucleotide) with various protein markers failed to identify the subcellular compartment targeted by the virus [8]. In the present study, we used pools of singly labelled FISH probes to specifically visualize the genomic RNA of lymphocytic choriomeningitis virus (LCMV), the prototypic mammarenavirus, with the goal of (i) defining the dynamics of genomic RNA replication during the course of acute infection, (ii) characterizing the subcellular localization of the genomic and antigenomic RNA, (iii) identifying the membrane-bound compartment targeted by arenavirus genomic RNA and (iv) describing how the virus may be taking advantage of this virus-targeted intracellular compartment.

The arenaviruses have a bisegmented genome, with each genomic segment encoding two genes in ambisense polarity [7]. The S genomic segment contains the negative-sense nucleoprotein (NP) gene and the pseudo-positive-sense glycoprotein precursor (GPC) gene (Fig. 1a) [7]. The Stellaris Probe Designer tool (Biosearch Technologies, Inc.) was used to design custom pools of 3' amine oligo FISH probes that would specifically hybridize to the S genomic or S antigenomic RNAs (Fig. 1a and Table S1, available in the online Supplementary Material). Probes were labelled post-synthesis with Cy3 or Cy5 dyes and purified as described previously [9]. To follow the replication dynamics of the S genomic and S antigenomic RNAs, we infected A549 cells with LCMV at an m.o.i. of 0.01, fixed infected cells as previously described [3] at the indicated times post-infection (p.i.), and performed FISH hybridization with S genome and S antigenome probes as previously described [10]. 3D datasets spanning the entire volume of the cells were acquired using a DeltaVision restoration microscopy system (GE Healthcare), and images were deconvolved using softWoRx software. Bright signal was observed in cells infected with LCMV, but very little signal was detected in uninfected cells, confirming that FISH probes specifically recognize the S genome and S antigenome (Fig. 1b, c). As expected, RNA signal was mainly observed in the cytoplasm, where S genome and S antigenome concentrated in cytoplasmic foci of varying size, brightness and subcellular localization (Fig. 1b, c). It is known that genomic and antigenomic RNA is encapsidated by the viral NP [7]. Thus, we stained for both the viral NP and the S genome to confirm their colocalization. For joint protein and RNA staining, we combined the immunofluorescence and FISH staining protocols as previously described [11]. NP and the S genome strongly colocalized in LCMV-infected cells (Fig. 1d). The line scan of the two fluorescent signals (shown in the inset in Fig. 1d) further confirms the colocalization between NP and the S genome (Fig. 1e).

Baird *et al*. [8] referred to foci of Tacaribe virus RNA colocalizing with viral NP as ‘replication-transcription complexes’. With the ability to label bona fide arenavirus genomic and antigenomic RNA, we next wanted to profile the composition and dynamics of these viral replication complexes. We first asked whether S genome and S antigenome traffic to distinct subcellular locations, or whether they remain associated in the same subcellular compartments. We therefore stained for both viral RNAs within the same cells and found that the S genome and S antigenome exhibit strong colocalization, supporting the idea that the genomic and antigenomic RNAs remain in close spatial proximity at the peak of acute infection (Fig. 1f, g).

**Fig. 2.**
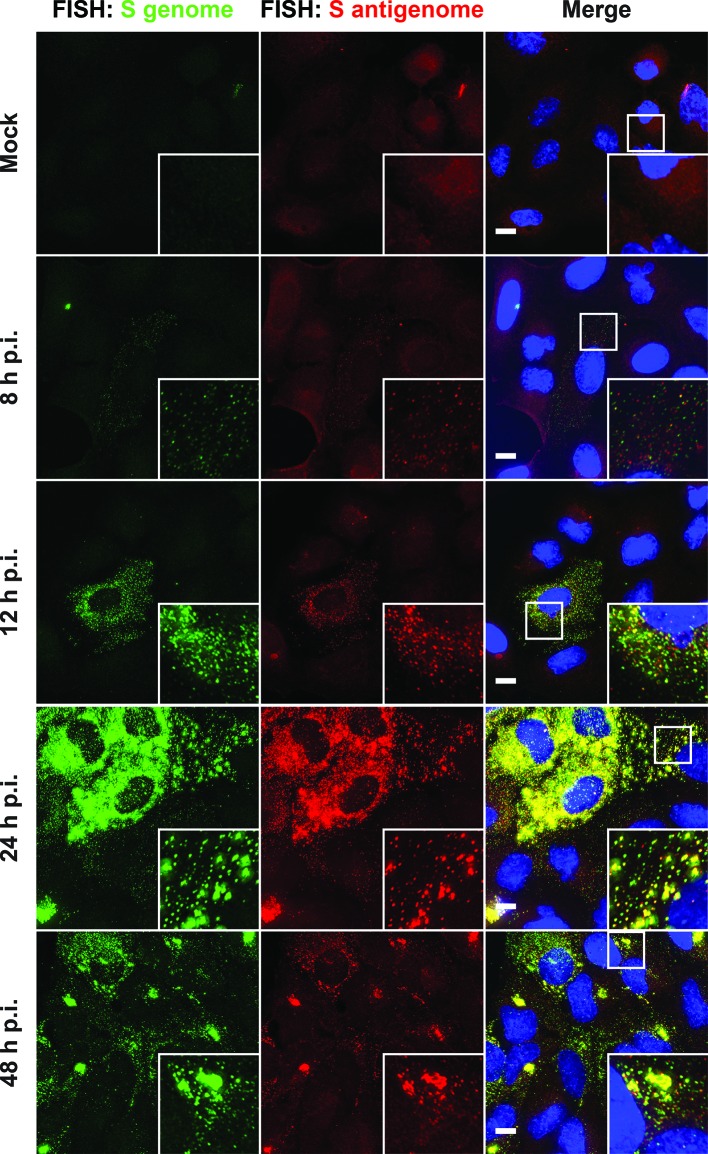
Dynamics of S genome and S antigenome during acute LCMV infection. Cells were infected with LCMV at an m.o.i. of 0.01 or not (mock) and fixed at multiple time points following infection. Maximum intensity projections of cells stained with S genome (Cy5) and S antigenome (Cy3) FISH probes are presented. Scale bar=10 µm.

To explore the temporal evolution of viral replication complexes, we next infected A549 cells with LCMV, fixed cells at different time points p.i., and stained for S genome and S antigenome (Fig. 2). At 8 h p.i., the S genome and S antigenome first became visible as small spots that were distributed throughout the cytoplasm, likely representing single viral genomes (Fig. 2). Interestingly, few of these individual genome and anti-genome signals co-localize, suggesting that clustering of viral RNAs occurs at a later stage during infection. At 12 and 24 h p.i., many cytoplasmic S genome/S antigenome foci were visible, and their size and intensity progressively increased, as well as the frequency of the co-localization of the genome and anti-genome signals (Fig. 2). At 48 h p.i., in many cells, the multiple bright cytoplasmic foci coalesced into one or a few large aggregates located adjacent to the nucleus (Fig. 2).

**Fig. 3.**
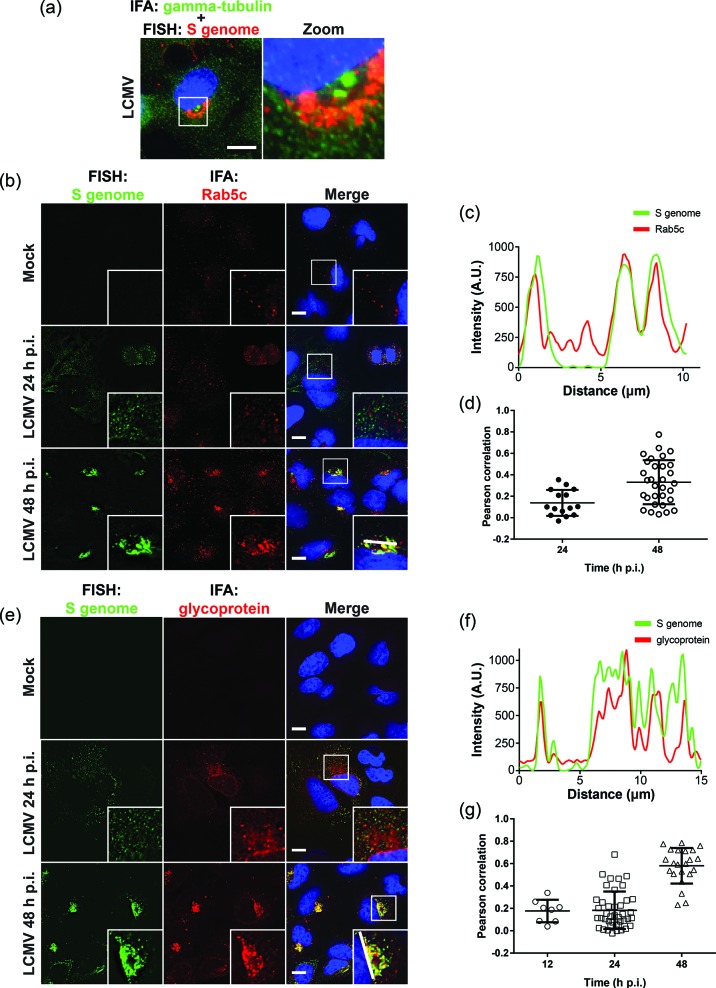
LCMV S segment genome selectively colocalizes with Rab5c and viral glycoprotein later during acute infection. (a) At 48 h p.i., perinuclear S genome aggregates localize near the microtubule organizing centre (MTOC), as visualized by gamma-tubulin [GTU-88, Sigma-Aldrich (primary); goat anti-mouse AlexaFluor 488 (secondary)] and S genome (Cy5-labelled FISH probes). A maximum intensity projection of a representative cell is shown. (b) Single Z stack of either mock- or LCMV-infected cells at the indicated time points after infection were stained for S genome (Cy5-labelled FISH probes) and Rab5c [sc-365667, Santa Cruz Biotechnology (primary); goat anti-rabbit AlexaFluor 488 (secondary)]. (c) Fluorescence line scan of S genome and Rab5c along the line indicated in the inset of the merged image at 48 h p.i. (b) is shown. (d) The Pearson’s correlation coefficient between the S genome and Rab5c fluorescence signals in individual infected cells at either 24 or 48 h p.i. was calculated in softWoRx software and the scores of individual cells were graphed. (e) Single Z stacks of either mock- or LCMV-infected cells that were stained for S genome (Cy5-labelled FISH probes) and viral glyocoprotein (GPC) [mouse anti-GPC, 33.6 (from M. Buchmeier, University of California Irvine) (primary) at 1 : 500; goat anti-mouse AlexaFluor 488 (secondary)] are shown. (f) A fluorescence line scan of S genome and GPC along the line indicated in the inset of the merged image at 48 h p.i. (e) is shown. (g) The Pearson’s correlation coefficient between the S genome and GPC fluorescence signals in individual infected cells at either 12, 24, or 48 h p.i. was calculated in softWoRx software and the scores of individual cells were graphed.

We were intrigued by the perinuclear localization of genomic RNA at the peak of acute infection and hypothesized that the arenavirus S genome RNA foci seen earlier in infection might be utilizing minus end-directed transport along microtubules to coalesce in larger structures near the cell’s microtubule organizing centre (MTOC). To test this, we stained cells for both gamma-tubulin (a marker of the MTOC) and S genome. Indeed, in most cases we found that the perinuclear S genome aggregate was located immediately adjacent to the MTOC (Fig. 3a). Previous observations that arenavirus ribonucleoprotein complexes copurified with cellular membranes [8], together with our observation that perinuclear S genome aggregates concentrate near the MTOC, led us to postulate that the S genome could be localizing to endosomal membranes and taking advantage of this organelle’s directed transport along microtubules [12]. It was previously demonstrated that Rab5c, an early endosomal marker [13], was required for the propagation of LCMV [14]. Rab5c is a Rab GTPase, a family of proteins that play critical roles in the establishment of vesicular identity, trafficking and effector protein recruitment [15]. Thus, we hypothesized that S genomic RNA may be localizing to Rab5c-positive membranes to promote some aspect of the LCMV life cycle. To test this, we stained cells infected with LCMV for either 24 or 48 h with an antibody specifically recognizing Rab5c and with FISH probes specific for S genome. Notably, at 48 h p.i., in a subset of cells, we observed increased levels of Rab5c and a perinuclear redistribution of this protein that resulted in strong colocalization with viral genome (Fig. 3b–d). However, the colocalization of S genome appeared highly time-dependent, as no colocalization was observed at 24 h p.i. (Fig. 3b–d). These data suggest that Rab5c may play an important role late in the LCMV life cycle, complementing previous work showing the importance of Rab5 for arenavirus entry [16, 17]. Furthermore, our observation of Rab5c’s involvement in the replication of arenaviral RNA is intriguing in light of other studies showing Rab5c as a cellular dependence factor for the replication of Zika virus, a flavivirus [18].

Our finding that Rab5c colocalizes with LCMV RNA was somewhat surprising given that previous work by Baird *et al*. did not observe any colocalization of Tacaribe virus replication/transcription complexes with endosomal markers, including Rab5a, which is closely related to Rab5c [8, 13]. The previous studies of Tacaribe virus examined a single time point after infection. Given the temporal specificity of the Rab5c/LCMV RNA association observed in the current study, it is possible that New World arenaviruses like Tacaribe do associate with endosomal markers, but that a kinetic study would be required to uncover such a result. Alternatively, it is possible that individual arenaviruses utilize different host machinery for genome replication and virus assembly. Indeed, related studies in our laboratory have demonstrated that replication of LCMV, but not the New World arenavirus Junín Candid #1, is impaired following siRNA silencing of Rab5c (C. M. Ziegler *et al.*, unpublished results). This result, which confirms the previously demonstrated importance of Rab5c for LCMV propagation [14], suggests that Old World arenaviruses such as LCMV, but not those of the New World lineage, are uniquely dependent upon Rab5c for successful completion of the life cycle. Further studies will be required to determine the extent to which Rab5c and other proteins in the Rab GTPase family are utilized by genetically diverse arenaviruses.

It is known that arenaviruses bud from the plasma membrane of infected cells [19]. Why, then, would LCMV S genome concentrate on the surface of Rab5c-positive vesicular structures in infected cells? One possibility is that these structures represent sites where different viral components pre-assemble before being trafficked together to the plasma membrane for budding. Indeed, it has been suggested that influenza A virus uses Rab11-positive membranes for the trafficking of viral ribonucleoproteins to the plasma membrane [20–22]. To test this possibility in the current system, we stained for another LCMV structural protein, its glycoprotein (GPC) (monoclonal antibody 33.6 [23]) and S genome at 12, 24 and 48 h p.i. We found that at 48 h p.i., in most cells there was a high degree of colocalization between GPC and S genome (Fig. 3e–g). As with Rab5c, the colocalization between these two viral components was variable at different stages of infection, and little colocalization was observed at earlier time points (Fig. 3e–g). As no direct NP–GPC interaction has been reported in the literature, GPC recruitment to encapsidated S genomic RNA would likely be dependent on the presence of the viral matrix protein, Z, which has been shown to interact with both NP and GPC [24, 25].

In summary, we describe the use of single-molecule resolution RNA FISH to specifically visualize LCMV S genome and S antigenome during the course of acute infection. For the first time, we reveal that the S genome and antigenome largely colocalize in the same subcellular structures during acute infection. Viral genomic RNA is highly dynamic during the course of acute infection, with many dim genomic RNA foci, likely representing individual viral genomes, progressively increasing in intensity and eventually coalescing into larger perinuclear structures, which, in many cells, appear to colocalize with the early endosomal marker Rab5c – shown by us and others to have a critical role in supporting the LCMV life cycle [14] (C. M. Ziegler *et al.*, unpublished results). We propose that LCMV is using this intracellular membrane as a scaffold for genome replication and possibly pre-assembly of viral components prior to being trafficked to the plasma membrane, where they will bud as infectious virions.

## Supplementary Data

58 Supplementary File 1
