# Supplementary material for: Visualization of the lymphocytic choriomeningitis mammarenavirus (LCMV) genome reveals the early endosome as a possible site for genome replication and viral particle pre-assembly
Source: J Gen Virol. 2017 Sep 27;98(10):2454–60. doi: 10.1099/jgv.0.000930 (PMC5725993; doi:10.1099/jgv.0.000930)
Supplement: Supplementary File 1 [file jgv-98-2454-s001.pdf]

Table S1. Full list and sequence of the FISH probes used in the current study.

Target	Probe Name	Probe Sequence (5' to 3')
LCMV S Genome	Genome 1	tttagaggcccaaatgttgt
LCMV S Genome	Genome 2	gctcccagatctgaaaactg
LCMV S Genome	Genome 3	cactcatggactgcatcatt
LCMV S Genome	Genome 4	tcgatgttgaaatgaccagg
LCMV S Genome	Genome 5	ggactcacagaataggaagg
LCMV S Genome	Genome 6	ccgatgacatcagaaagctt
LCMV S Genome	Genome 7	atggttctaagctgtcaagg
LCMV S Genome	Genome 8	tcgtcagttataggtgctct
LCMV S Genome	Genome 9	gatcttgccgacctcttcaa
LCMV S Genome	Genome 10	gattccaagtactcacacgg
LCMV S Genome	Genome 11	ggaacccgttgatcaaaaac
LCMV S Genome	Genome 12	cgggcagttcatacactttt
LCMV S Genome	Genome 13	tgatccagtggaaatagcaa
LCMV S Genome	Genome 14	caacgctcctacatggattg
LCMV S Genome	Genome 15	tacagccagacaatgctttt
LCMV S Genome	Genome 16	agaaacctgcagtcaattca
LCMV S Genome	Genome 17	gcatgggaaaacacaacaat
LCMV S Genome	Genome 18	gatggccatacatagcttgt
LCMV S Genome	Genome 19	aaagtttgcctttcaggtga
LCMV S Genome	Genome 20	caggaacccttatgaaaaca
LCMV S Genome	Genome 21	ttgtttcagaccaagttggg
LCMV S Genome	Genome 22	ggccaagagaaaactcaaca
LCMV S Genome	Genome 23	gacctcttgaaggcagttct
LCMV S Genome	Genome 24	ttttgatcaagccaagcaac
LCMV S Genome	Genome 25	aactttagtcttggtgctgc
LCMV S Genome	Genome 26	gtcatcactgaacagcagtc
LCMV S Genome	Genome 27	tcttgaaaggctgaaagaca
LCMV S Genome	Genome 28	ggcttgctttacacagtcaa
LCMV S Genome	Genome 29	tcaatgacgttgtacaagcg
LCMV S Genome	Genome 30	ctatggcttgtatggccaaa
LCMV S Genome	Genome 31	aatcaatttggcacaatgcc
LCMV S Genome	Genome 32	gggatgtgaaagactcatca
LCMV S Genome	Genome 33	ttgggatgagaaagcctcag
LCMV S Genome	Genome 34	gaccaaagatctcagatcct
LCMV S Genome	Genome 35	gggaacttaacaacacagca
LCMV S Genome	Genome 36	ccaggcttcaggggtatata
LCMV S Genome	Genome 37	ccaagatcatgaggtctgaa
LCMV S Genome	Genome 38	gctgaccttgagaagctgaa
LCMV S Genome	Genome 39	cagtgcagaagaactgatgt
LCMV S Genome	Genome 40	actgtacattctcttgtgga
LCMV S Genome	Genome 41	gagactcagaagtctcaacc
LCMV S Genome	Genome 42	aagagagatgacaaagacct
LCMV S Genome	Genome 43	cttctctgaggtcagcaatg
LCMV S Genome	Genome 44	ccaaccttctgaatgggttg

LCMV S Genome	Genome 45	ggctgctgtcattaaggatg
LCMV S Genome	Genome 46	gcagagcttcacatcagatg
LCMV S Genome	Genome 47	cgcaagcattgagaagagaa
LCMV S Genome	Genome 48	aggaagttaagagcttccaa
LCMV S Antigenome	Antigenome 1	ggtgtaaaaaccgtctggaa
LCMV S Antigenome	Antigenome 2	accgattaaccaacaaagga
LCMV S Antigenome	Antigenome 3	ataaaaggtggctcatgtcc
LCMV S Antigenome	Antigenome 4	ctagtcagcatcttcctgca
LCMV S Antigenome	Antigenome 5	gatgttttccacatctgcat
LCMV S Antigenome	Antigenome 6	ccctagcattgatggacctt
LCMV S Antigenome	Antigenome 7	aacaggaagccgataacatg
LCMV S Antigenome	Antigenome 8	aaatgagacccacttcagtg
LCMV S Antigenome	Antigenome 9	gcttgtcaccaatggttctt
LCMV S Antigenome	Antigenome 10	gaaactagtgtccccaagtg
LCMV S Antigenome	Antigenome 11	acctagaacatgcaaagacc
LCMV S Antigenome	Antigenome 12	gggtgccatattgcaattac
LCMV S Antigenome	Antigenome 13	actactgatgaggaaccact
LCMV S Antigenome	Antigenome 14	gtagaatctgccttgcactt
LCMV S Antigenome	Antigenome 15	aggctgctttgagtaagttc
LCMV S Antigenome	Antigenome 16	gctgcgactaattgactaca
LCMV S Antigenome	Antigenome 17	tcatgatgccgaattctgtg
LCMV S Antigenome	Antigenome 18	cagttgcgaaatgcaatgta
LCMV S Antigenome	Antigenome 19	gcttaagtgtttcgggaaca
LCMV S Antigenome	Antigenome 20	aatggatgattcttgctgca
LCMV S Antigenome	Antigenome 21	agaatccaggtggttattgc
LCMV S Antigenome	Antigenome 22	acattcacctggactttgtc
LCMV S Antigenome	Antigenome 23	tcttcactaggagactagcg
LCMV S Antigenome	Antigenome 24	tctcctttcccaagagaaga
LCMV S Antigenome	Antigenome 25	acatatgcaggtccttttgg
LCMV S Antigenome	Antigenome 26	atagaacctgggaaaaccac
LCMV S Antigenome	Antigenome 27	gccagacgagttaccaatac
LCMV S Antigenome	Antigenome 28	atgtttagaactgccttcgg
LCMV S Antigenome	Antigenome 29	agtgtagaaccttcagaggt
LCMV S Antigenome	Antigenome 30	ctcagatcgacaaagtgctc
LCMV S Antigenome	Antigenome 31	cagtatcctgcgacttcaac
LCMV S Antigenome	Antigenome 32	tcagagggaactccaactat
LCMV S Antigenome	Antigenome 33	tagtttcgagcctacacctc
LCMV S Antigenome	Antigenome 34	tttgaccacacactcatgag
LCMV S Antigenome	Antigenome 35	ttttgcaatctgacctctgc
LCMV S Antigenome	Antigenome 36	agaattgaccttcaccaatg
LCMV S Antigenome	Antigenome 37	acatcagtatggggacttct
LCMV S Antigenome	Antigenome 38	gcatgttcagccaacaactc
LCMV S Antigenome	Antigenome 39	cacatctgaacctgaccatg
LCMV S Antigenome	Antigenome 40	ccaatttaagtcagtggagt
LCMV S Antigenome	Antigenome 41	acccgacatttacaaaggag
LCMV S Antigenome	Antigenome 42	gtggcatgtacggtcttaag
LCMV S Antigenome	Antigenome 43	cagtttcctacttctggctg

LCMV S Antigenome	Antigenome 44	cctgtgggatattcgcattg
LCMV S Antigenome	Antigenome 45	aaggctgtctacaattttgc
LCMV S Antigenome	Antigenome 46	ttgtgcttatcgtgatcacg
LCMV S Antigenome	Antigenome 47	ctcacatcatcgatgaggtg
LCMV S Antigenome	Antigenome 48	gtgacaatgtttgaggctct
